# Initial experience of a novel ureteral silicon-covered metallic mesh stent in malignant ureteric obstruction: a single-center retrospective study

**DOI:** 10.1186/s12894-024-01653-y

**Published:** 2024-12-19

**Authors:** Chung Un Lee, Seol Ho Choo, Jae Hoon Chung, Deok Hyun Han

**Affiliations:** 1 Department of Urology, Chung-Ang University Gwangmyeong Hospital, Chung-Ang University College of Medicine, Gwangmyeong, Gyeonggi-do Republic of Korea; 2 Department of Urology, Ajou University School of Medicine, Suwon, Korea; 3 Department of Urology, Hallym University Sacred Heart Hospital, Hallym University College of Medicine, Anyang, Korea; 4 Department of Urology, Samsung Medical Center, Sungkyunkwan University School of Medicine, 81 Irwon-ro, Gangnam-gu, Seoul, 06351 Korea

**Keywords:** Malignant ureteric obstruction, Metallic ureteric stent, Ureteral stent, Retrograde manner, Clinical outcome

## Abstract

**Background:**

This study aimed to investigate initial experiences and outcomes of a retrograde inserted new silicone-covered metallic ureteric stent (Urexel™) for malignant ureteric obstruction.

**Methods:**

We retrospectively reviewed the medical records for 12 consecutive patients who underwent Urexel™ stent placement for malignant ureteric obstruction from March 2020 to March 2021. The Urexel™ stent is a segmental metallic ureteric stent composed of a nitinol mesh covered with a silicone membrane. We evaluated the clinical outcomes and complications of this new metallic ureteric stent.

**Results:**

The median age of patients was 61.5 (44–82) years, and the median follow-up was 25.5 (4–37) months. One of the 12 patients underwent bilateral stent insertion, Urexel™ stents were placed in a total of 13 ureteral units. There was no technical failure during stent placement. The median length of obstructions was 9 (1–22) cm. Balloon dilation was necessary in 38.5% (5/13 ureter units) of cases. The 6-month success rate was 83.3%, 1-year success rate was 70%, and estimated success rate was 44.4% at 2-year. Encrustation, migration and hyperplasia were the cause of overall treatment failure in all 6 cases of failure, with median elapsed time to failure of 9.5 (1–30) months. Common complications included persistent pain, acute pyelonephritis, and lower urinary tract symptoms, but they were Clavien-Dindo grade I or II.

**Conclusions:**

In this initial series of novel ureteral silicon-covered metallic mesh stents, Urexel™ provided acceptable success and complication rate in malignant ureteric obstructions.

## Background

Malignant ureteric obstruction (MUO) is frequent with gynecological and colorectal malignancies, and the median survival is short at 6–8 months [1, 2]. Purposes of MUO management are symptom relief and sparing renal function. Various interventions are applicable, but the current primary management for MUO is ureteric stent insertion or percutaneous nephrostomy (PCN) [3]. Traditional polymer double-J stent insertion is an effective and acceptable treatment option but has limitations such as requirement of regular stent exchange and easily rendered non-functional due to tumor ingrowth or encrustation [4, 5]. PCN is more effective and reliable than double-J stents, but retaining PCN is very bothersome for patients and PCN catheter is very easy to migrate, and self-removal of the catheter frequently occurs, especially in patients who have altered mental capability and/or physical mobility [6].

Many metallic ureteric stents have been developed to overcome the limitations of conventional PCN or double-J stents. Previously developed ureteric uncovered metallic mesh stents tend to be easily obstructed by urothelial hyperplasia or tumor ingrowth [7, 8]. To complement this, Allium^®^ URS, Memokath™-051, Resonance^®^, and Uventa™, are introduced and currently available metallic ureteric stents. Metallic mesh stents have the advantage of being semipermanent compared to existing metallic ureteric stents and help with peristalsis of the ureter because they are segmental stents that do not span the entire ureteral length [9, 10]. Among the products mentioned above, metallic mesh stents are Allium^®^ URS, Memokath™-051, and Uventa™.

Despite the introduction of the ureteric covered metallic mesh stent to compensate for the shortcomings of the existing ureteric uncovered metallic mesh, each metallic mesh stent has limitations according to their material and structures. Recently, one group reported favorable results for a newly developed silicone-covered metallic ureteric stent (Urexel™ stent, S&G Biotech, Yongin, Korea) inserted in an antegrade manner though the PCN tract for MUO patients [11].

There were no studies about novel silicone-covered metallic mesh stent inserted in a retrograde manner and in this study, we evaluated the initial outcomes of this novel silicone-covered metallic mesh stent when it is placed retrograde in MUO patients.

## Methods

### Study population

This study was approved by the Institutional Review Board of the Samsung Medical Center (IRB No. 2021-10-090), which waived the requirement for informed consent owing to the retrospective nature of this study. All the study protocols were performed in accordance with the principles of the Declaration of Helsinki.

We retrospectively reviewed the medical records of 12 consecutive patients (three male and nine female) who underwent Urexel™ ureteric stent placement because of MUO from March 2020 to March 2021. Patients were fully explained about the possibility of failure before undergoing the procedure, and the procedure was performed with consent. However, there were no cases of failure during the procedure.

All patients were preoperatively evaluated with a medical history, physical examination, and imaging studies including excretory urography, computerized tomography, retrograde pyelography, and nuclear renography. The Urexel™ stent placement was decided after dedicated counselling about risks and benefits, as well as comprehensive discussion about alternative therapeutic options.

### Material and operative procedures

**Fig. 1:**
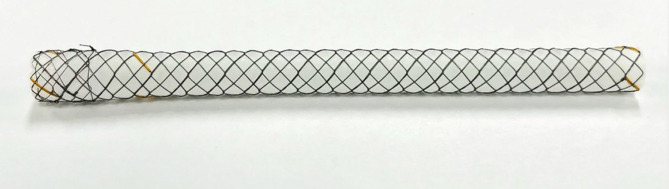
Feature of Urexel™ stent - a segmental metallic stent composed of a nitinol mesh and is fully covered with a silicone membrane

The Urexel™ stent is a segmental metallic ureteric stent composed of a nitinol mesh and is fully covered with a silicone membrane (Fig. 1). The stent is distally tapered with a 1-mm difference in diameter between proximal and distal ends. For preventing stent migration, an additional external mesh that is not covered with silicone was added at the proximal 1 cm of the outer surface. The stent length varied from 6 cm to 16 cm with an external diameter of 7 mm [11, 12].

**Fig. 2:**
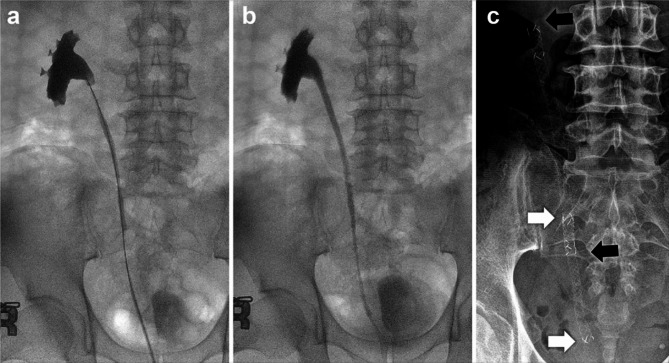
Placement of multiple Urexel^TM^ stent with overlapping of more than 2 cm to reinforce the radial force and prevent migration. **a. **Retrograde pyelogram demonstrating multiple ureteric obstruction.** b. **Ureteral balloon dilation performed prior to stent placement **c. **Co-axial placement of double Urexel^TM^ stents with approximately 2cm overlap

Stent placement was performed in a retrograde fashion in all patients by a single surgeon who had experienced more than 250 cases of metallic stent placement using cystoscopy and/or ureteroscopy under general anesthesia. A retrograde pyelogram was performed to assess the location and length of ureteric obstructions. In cases of severe obstruction, ureteral balloon dilation was employed before stent placement (A 6 mm with 10 cm length BARD X-Force, Bard Medical, Atm-until completion of ballooning up to 30 atm). And then, insert the guide wire sufficiently beyond the obstruction area. Enter the delivery system along the guide wire, deploy the stent in the area where we want to install the stent, and remove the delivery system. As the delivery system is removed, the stent expands naturally. We inserted stents at least 2 cm longer than the total obstruction length to cover sufficient normal ureter at each distal and proximal end. In cases that required multiple stents because of long or multiple strictures, the stents were overlapped by more than 2 cm to reinforce the radial force and to prevent migration (Fig. 2). For removal, simply hold the tip of the stent with the forceps and pull.

### Outcome assessment

For evaluation of stent patency, imaging studies including diuretic renography and abdominal computed tomography were used at baseline and every three months after stent placement. Success was defined as no obstruction on static or functional imaging study with no additional interventions. Success was assessed at 6 months, 1 year, 2 years. Stent-related complications were evaluated every follow-up visit according to the modified Clavien-Dindo classification [13]. And in case that needed stent removal, procedure-related complication was also evaluated.

### Statistical analyses

Descriptive statistics included the frequencies and proportions of categorical variables. Continuous variables are presented as median (range). Kaplan–Meier survival analysis was used to illustrate the success rate after Urexel™ installation. All statistical analyses were performed using IBM SPSS^®^ (version 27.0; SPSS Inc., Chicago, IL, USA).

## Results

The median age of patients was 61.5 (44–82) years, and the median follow-up was 25.5 months with a range of 4–37 months. Urexel™ stents were placed in a total of 13 ureteral units in 12 patients. One patient underwent bilateral stent placement. The underlying causes of ureteric obstructions were colorectal cancer (4/12), gynecologic cancer (4/12), stomach cancer (3/12), and breast cancer (1/12). There was a history of previous radiation therapy in 66.7% (8/12) of cases. The median length of obstruction was 9 (1–22) cm (Table 1).

**Table 1:** Baseline characteristics of patients with Urexel™

	*N* = 12
**Age at surgery**,** year (range)**	61.5 (44–82)
**Sex**,** n (%)**	
Male	3 (25)
Female	9 (75)
**Ureter units**,** n**	13
Location of ureteral obstruction, n (%)	
Proximal	1 (7.7)
Distal	1 (7.7)
Proximal to middle	3 (23.1)
Proximal to distal	2 (15.4)
Middle to distal	6 (46.2)
**Laterality**,** n (%)**	
Right	8 (66.7)
Left	3 (25)
Bilateral	1 (8.3)
**Underlying cause of ureteric obstruction**,** n (%)**	
Colorectal cancer	4 (33.3)
Gynecologic cancer	4 (33.3)
Stomach cancer	3 (25)
Breast cancer	1 (8.3)
**Previous percutaneous nephrostomy**,** n (%)**	4 (33.3)
Unilateral	4 (100)
**Previous double-J stent**,** n (%)**	7 (58.3)
Unilateral	6 (85.7)
Bilateral	1 (14.3)
**Preoperative creatinine**,** mg/dL (range)**	0.73 (0.53–3.41)
**Preoperative eGFR**,** mL/min/1.73m**^**2**^**(range)**	87.2 (14.8-119.1)
**Previous radiation history**,** n (%)**	8 (66.7)
**Length of obstruction**,** cm (range)**	9 (1–22)
**Total follow-up**,** months (range)**	25.5 (4–37)

There was no technical failure during stent placement. Two stents were inserted coaxially in 53.8% (7/13) of units, and 16 cm length of stent was most commonly used (84.6%, 11/13). Pre-ballooning using 18Fr. balloon was performed before Urexel™ stent placement in 38.5% (5/13), and the stent was placed across the ureterovesical junction in 92.3% (12/13) of cases.

**Fig. 3:**
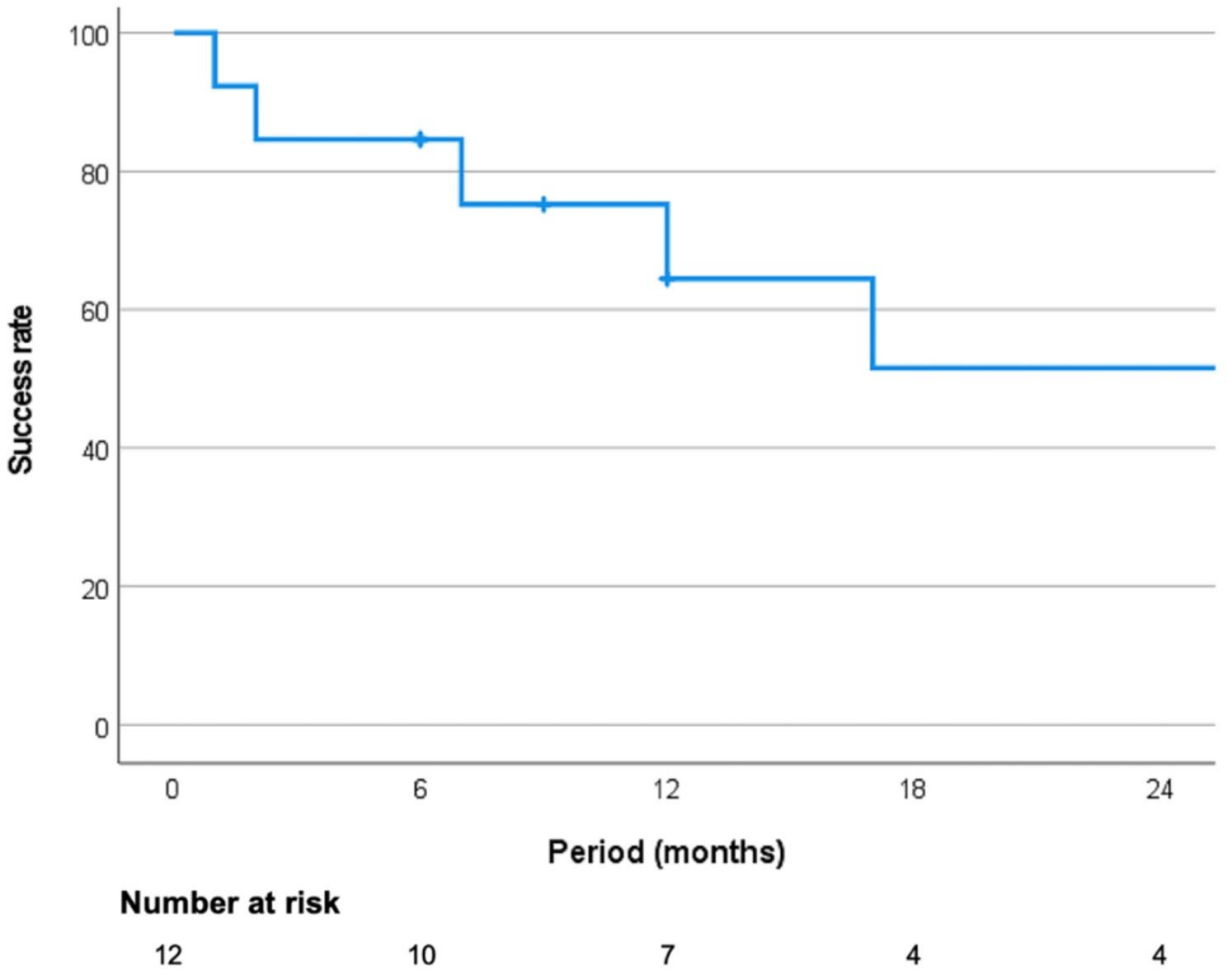
Kaplan-Meier success rate curve of patients with Urexel™

In seven ureters units (53.8%), stent could relieve ureteral obstruction without any intervention during total follow up. Success rate for patients at 6 months, 1 year, and 2 years were 83.3% (10/12), 70% (7/10), and 44.4% (4/9) respectively. Five patients died during follow-up, and three of them were able to resolve ureteral obstruction without additional treatment after insertion of Urexel™ until death. Kaplan-Meier curve for success rate for patients was shown at Fig. 3.

Most common reason of stent failure were migration combined with encrustation and encrustation alone 15.4% (2/13) that was followed by migration alone and hyperplasia 7.7% (1/13), and all patients with stent failure showed more delayed excretion pattern in renal scan, higher creatinine levels and lower eGFR compared to baseline (Table 2). In all units with failure, Urexel™ stents were removed successfully by endoscopic procedures. In three units, double-J stent (Inlay Optima^tm^, Bard Medical) was placed after Urexel removal. In two units, percutaneous nephrostomy was needed. In one unit in which Urexel™ was failed due to migration combined with encrustation, ureteral obstruction was resolved, and no additional procedure was necessary. There was one unit of acute pyelonephritis after endoscopic Urexel™ removal. And there was no other procedure-related complication during stent removal.

**Table 2:** Surgical outcomes of patients with Urexel™ according to ureter units

	*N* = 13
**Number of stents,** ***n*** **(%)**	
1	6 (46.2)
2	7 (53.8)
**Length of the stent-indwelled segment**,** n (%)**	
6 cm	4 (30.8)
8 cm	4 (30.8)
14 cm	1 (7.7)
16 cm	11 (84.6)
**Balloon dilation before stent insertion**,** n (%)**	5 (38.5)
**Stent placed across the ureterovesical junction**,** n (%)**	12 (92.3)
**Postoperative creatinine**, mg/dL (range)^*^	
Postoperative 1-month creatinine	0.69 (0.57–1.48)
Postoperative 3-month creatinine	0.77 (0.48–4.14)
**Postoperative eGFR**,** mL/min/1.73m**^**2**^(range)^*^	
Postoperative 1-month eGFR	96.4 (40.7-112.4)
Postoperative 3-month eGFR	89.4 (10.4-122.2)
**Success rate**, %^*^	
6-month success rate	83.3
1-year success rate	70
2-year success rate	44.4
**Reason for failure**,** n (%)**	
Migration & encrustation	2 (15.4)
Encrustation	2 (15.4)
Migration	1 (7.7)
Hyperplasia	1 (7.7)

* These values are based on the number of patients (*N* = 12)

The Clavien-Dindo grade III complication rate was 25% (3/12) in patients and 23.1%, (3/13) in ureter units. Stent migration occurred in three patients and units, and encrustation was combined in two patients and units. Other grade I or II complications included persistent pain that required analgesics (2/12 patients, 3/13 units), acute pyelonephritis (2/12 patients, 2/13 units), and lower urinary tract symptoms (2/12 patients, 2/13 units). There was no grade IV or V complication (Table 3).

**Table 3:** Complications of patients with Urexel™ according to individuals and ureter units

	*N* = 12	*N* = 13
**Clavien-Dindo grade I**,** n (%)**	2 (16.7)	2 (15.4)
Lower urinary tract symptoms	2 (100)	2 (100)
**Clavien-Dindo grade II**,** n (%)**	4 (33.3)	5 (38.5)
Required analgesics	2 (50)	3 (60)
Pyelonephritis	2 (50)	2 (40)
**Clavien-Dindo grade III**,** n (%)**	3 (25)	3 (23.1)
Stent migration with encrustation	2 (66.7)	2 (66.7)
Only stent migration	1 (33.3)	1 (33.3)
**Clavien-Dindo grade IV or V**,** n (%)**	0 (0)	0 (0)

## Discussion

Long-standing ureteric obstructions significantly compromise renal function. Alleviating the obstruction is essential for pain relief, reduction of infection risk, and restoration of adequate renal function. Surgical urinary diversions including open nephrostomy, enteral conduits, cutaneous ureterostomy, and enteric substitution are not always indicated because of underlying disease and/or co-morbidities. They are also often technically difficult and can cause many complications [3]. Conventionally, polymeric stent or PCN were employed, but numerous complications and/or negative impact on patient quality of life distressed many patients. Ureteric metallic stents were introduced as a less invasive and more long-lasting treatment without frequent stent changes for ureteric obstructions and would be suitable for patients who are not fit for further oncologic treatment or frequent anesthesia. Patients who wish to avoid PCN or frequent stent change would also be good candidates for metallic stent. Various types of ureteric metallic stents have been developed, and each has benefits and limitations [14, 15].

The Urexel™ stent is a recently developed self-expanding segmental metallic stent. There are some previous reports have shown that Urexel™ provided favorable results in MUO [11] and ureteric stricture following kidney transplantation [12] when placed in an antegrade manner. Kim et al. [11] enrolled 19 MUO patients for prospective comparison of Urexel™ (10/19) and double-J ureteral stent (9/19). The Urexel™ group showed higher success rate at three months (90% vs. 35%) and at six months (57% vs. 21%), with a higher overall success rate (*p* = 0.041). Stent migration occurred in one patient in the Urexel™ group at one month after stent placement. Recently, a retrospective study was reported comparing ureter unit (54/84) in Urexel™ and (30/84) ureter unit in double-J ureteral stent for MUO in advanced gastric cancer, and the cumulative success rates at 1, 3, 6, and 12 months were 77%, 74%, 70%, and 70%, in the Urexel™ group and 72%, 60%, 53%, and 26%, in the double-J group, reporting that Urexel™ group is better in maintaining patency [16]. Tsauo et al. reported a retrospective study with 175 ureteral units treated with Urexel™ in an antegrade manner for MUO, and stent malfunction was found to be 21.4% (37 ureteral units) during the follow-up period. Through this, it was shown that Urexel™ placement was effective [17]. Cao et al. [12] reported the outcomes of temporarily placed Urexel™ in eight patients with refractory ureteral stricture following kidney transplantation. Stent migration occurred in two patients (28.6%), and the overall success rate after stent removal was 71.4% (5/7) during a mean 22.6 months of follow-up.

In addition to Urexel™, research on other ureteral metal stents cannot be overlooked. In the case of Resonance^®^, the success rate at 6 months is reported to be 77.3% and at 12 months is 70.3% [18], and for UROSOFT tumor stent, the success rate at 5 months is reported to be 52% [5]. In this study, the 6-month success rate is reported as 83.3% and the 12-month success rate is reported as 70%, and the results are comparable to the previous studies. However, the ureteral stents mentioned above are stents that require periodic replacement, so their use concept is different from Urexel™.

Retrograde ureteral stent placement has several advantages over antegrade insertion. It does not require nephrostomy, so there is no potential risk related with percutaneous access such as pleural complication, internal solid organ injury, and significant bleeding. Retrograde placement enables an endoscopic procedure, so concomitant procedures including diagnostic endoscopy, biopsy of abnormal tissue, and stent removal are possible. Further, when stent location is not favorable, the position of the distal stent is easily adjustable with retrograde procedures. Above all, it is more familiar to a urologist. There are no data demonstrating the clinical outcomes of retrograde Urexel™ stent placement; the current study aims to address this lack.

Stent migration is one of the most hindersome complications in metallic stent placement [3]. Soft strictures, insufficient anchorage, and propulsion by antegrade peristalsis were considered as contributing factors for migration [19]. In the early days of metallic mesh stent use, tissue ingrowth was a major cause of failure [20]. To overcome this, a covered metallic mesh stent has been used, although it did not address the issue of migration [21]. Thereafter, the Uventa™ stent was introduced and showed favorable results in terms of migration [15, 22, 23]. However, the mesh exposed to the outside of the PTFE cover adhered to the ureteral walls, and stent removal is difficult [24].

A metallic ureteral stent mainly is used to avoid frequent stent change to achieve long-term or permanent indwelling. However, in cases of complications including stent malfunction, migration, recurrent urinary tract infection, and intractable stent-induced pain, the metallic ureteral stent should be removed. This indicates the need for feasibility of stent removal or exchange. Most of the currently available metallic stents have their own mechanism to facilitate stent removal [25–27]. In consequence, although migration and removability are opposing requirements, there is need for a stent that offers both. Urexel™ has a 1-cm-long additional segment with external mesh that is not covered by silicone. We expect this distinct design to contribute to preventing stent migration. In this study, stent migration was observed in 23.1% (3/13) of cases and was the major cause of overall failure. This is similar to cases with other metallic stents such as Allium^®^ URS (14%) [25] and Memokath™-051 (20%) [15, 20, 28, 29]. Stent migration was observed in three of the initial six cases and not at all in the later six cases. The surgeon’s experience with Urexel™ stent is one explanation for this finding, but the sample size of the study was not large enough to make any conclusions.

The metallic mesh of Urexel™ is not exposed except for 1 cm at both ends and is surrounded by a silicon membrane to prevent adhesion to ureteral walls and reduce stone formation around the stent. When Urexel™ is pulled from the end, the lumen narrows. These features prevent attachment to the ureteral wall and aid in easy removal. Stent removal was necessary in six patients with Urexel™ placement, and there was no adverse event during stent removal. Complications did not exceed Grade 3b, and no serious complication such as fistula formation was observed. The possibility of stent removal via either antegrade or retrograde method was also demonstrated in a previous report about temporary Urexel™ indwelling in eight kidney transplanted patients [12]. Two characteristics of the Urexel™ foster easy endoscopic removal. The nitinol mesh shrinks and becomes more flexible when cold saline is applied. The silicone membrane prevents ingrowth of tissue into the lumen of the stents.

The economic aspect should also be taken into consideration. Several studies have already reported the cost-effectiveness of the less repeat procedure of metal stents compared to repeated replacement of polymer ureteral stents [30, 31]. Urexel is a ureteral stent that is intended for permanent placement and is expected to provide greater economic value compared to existing metal stents. In addition, expectations are even higher in that it can minimize patient discomfort caused by ureteral stent replacement.

Our study has some limitations including its retrospective nature. The sample size was small and the follow-up period was insufficient. We could not evaluate statistical difference but could offer a descriptive report. However, we think our study showed acceptable initial outcomes with a new metallic ureteric stent for MUO. The Urexel™ stent could have a role in symptom relief and maintenance of renal function for MUO patients.

## Conclusion

In this initial series, a novel silicon-covered metallic mesh stent, Urexel™, showed favorable clinical outcomes. The stent provided acceptable success and complication rates in malignant ureteric obstruction. Although the role of a metallic ureteric stent for MUO remains undefined, the Urexel™ stent would be a good candidate as an effective and safe internal urinary diversion modality.

## Data Availability

The datasets used and analyzed during the current study are available from the corresponding author on reasonable request.

## Abbreviations

MUO: Malignant ureteric obstruction
PCN: Percutaneous nephrostomy
IRB: Institutional Review Board
